# Isosteviol sodium protects the cardiomyocyte response associated with the SIRT1/PGC‐1α pathway

**DOI:** 10.1111/jcmm.15715

**Published:** 2020-08-05

**Authors:** Ying Mei, Bo Liu, Hao Su, Hao Zhang, Fei Liu, Qingjin Ke, Xiaoou Sun, Wen Tan

**Affiliations:** ^1^ Institute of Biomedical and Pharmaceutical Sciences Guangdong University of Technology Guangzhou China

**Keywords:** cardiomyocyte dysfunction, isosteviol sodium, mitochondria, PGC‐1α, SIRT1

## Abstract

Cardiomyocyte dysfunction is attributed to excess oxidative damage, but the molecular pathways involved in this process have not been completely elucidated. Evidence indicates that isosteviol sodium (STVNa) has cardioprotective effects. We therefore aimed to identify the effect of STVNa on cardiomyocytes, as well as the potential mechanisms involved in this process. We established two myocardial hypertrophy models by treating H9c2 cells with high glucose (HG) and isoprenaline (ISO). Our results showed that STVNa reduced H9c2 mitochondrial damage by attenuating oxidative damage and altering the morphology of mitochondria. The results also indicated that STVNa had a positive effect on HG‐ and ISO‐induced damages via mitochondrial biogenesis. The protective effects of STVNa on cardiomyocytes were associated with the regulation of the SIRT1/PGC‐1α signalling pathway. Importantly, the effects of STVNa involved different methods of regulation in the two models, which was confirmed by experiments using an inhibitor and activator of SIRT1. Together, the results provide the basis for using STVNa as a therapy for the prevention of cardiomyocyte dysfunctions.

## 1 INTRODUCTION

Cardiac hypertrophy, the first phase of cardiovascular disease, induces heart failure.^1^ It is associated with increased interstitial fibrosis, cell death and cardiac dysfunction,^2^ and is characterized by up‐regulation of genes such as *ANP*, *BNP* and *β‐MHC*. In addition, increased cell area is an important characteristic of cardiac hypertrophy. During this process, many signalling pathways play important roles, such as Akt (threonine kinase), ERK (extracellular signal‐regulated kinase) and Ca^2+^/calmodulin‐dependent protein kinase II.^3^, ^4^, ^5^ Protecting the viability and function of cells under conditions of oxidative stress is of great importance in the prevention and treatment of cardiac hypertrophy. Furthermore, high glucose (HG) and isoprenaline (ISO) are traditional methods used to induce cardiac hypertrophy, although their molecular mechanisms differ.

SIRT1 (silent information regulator transcript‐1), an NAD^+^‐dependent nuclear histone deacetylase, regulates the activity of histone deacetylase by deacetylation of a variety of histones, which act as substrates. It participates in multiple metabolic pathways, such as the regulation of mammalian cell life signals, metabolism and insulin secretion. SIRT1 also plays an important role in metabolic syndrome, cell apoptosis, cardiovascular diseases and neurodegenerative diseases,^6^, ^7^, ^8^ and is an activator of peroxisome proliferator‐activated receptor gamma coactivator 1‐alpha (PGC‐1α).^7^ Recently, studies have reported that SIRT1 contributes to cardiac metabolism by activating PGC‐1α and pyruvate dehydrogenase kinase 4 (PDK4) expressions during cardiac hypertrophy.^9^, ^10^, ^11^ Considering the profound impact of SIRT1 on mitochondrial biogenesis and metabolism, characterizing the link between regulation of SIRT1 and HG or ISO exposure is a key issue in identifying the molecular mechanisms involved in cardiac damage induced by HG and ISO.

Isosteviol, isolated from the herb, Stevia rebaudiana, is a widely known sweetener.^12^ It has been reported to have many pharmacological functions, such as anti‐inflammatory, anti‐tumour, and neuroprotective effects.^13^, ^14^ Isosteviol sodium (STVNa) is a sodium salt of isosteviol, and recent studies have found that it has strong effects on cardiomyocytes.^15^, ^16^ However, the underlying mechanisms of the cardiac hypertrophy effect of STVNa are still not completely clear. The present study was therefore designed to identify the effect of STVNa on cardiomyocytes, as well as the potential mechanisms involved.

## 2 MATERIALS AND METHODS

### 2.1 Reagents and chemicals

Isosteviol sodium powder was provided by the Chemical Development Laboratories of Key Biological Pharmaceutical Company. The 2^'^,7^'^‐dichlorofluorescein diacetate (DCFH‐DA), JC‐1 and phalloidin were purchased from Sigma‐Aldrich. The antibodies against PGC‐1α were purchased from Abcam. SIRT1 and AMPK antibodies were obtained from Santa Cruz Biotechnology. Secondary antibodies were purchased from Affinity Biosciences.

### 2.2 Cell culture and treatment

The H9c2 cells were purchased from the Type Culture Collection of the Chinese Academy of Sciences (Shanghai, China) and cultured in Dulbecco's modified Eagle's medium (DMEM)/low glucose in an atmosphere of 5% CO_2_ at 37°C. DMEM contained 10% foetal bovine serum, 100 U/mL penicillin and 100 mg/mL streptomycin.

### 2.3 Cell viability assay

Cell viability was assessed using the Cell Counting Kit‐8 (CCK8) assay (Beyotime). First, H9c2 cells were seeded in a 96‐well plate. When the cells reached 70% confluency, they were treated with HG or ISO with or without STVNa for 48 hours. Then, the CCK‐8 diluent (0.5 mg/mL) was added at a volume of 100 μL per well. Lastly, the optical density was measured at 450 nm after incubation for 1‐4 hours at 37°C.

### 2.4 Assessment of intracellular reactive oxygen species (ROS)

H9c2 cells were seeded in 24‐well plates. When the cells reached 70% confluency they were treated according to the experimental design. Intracellular ROS accumulation was assessed using DCFH‐DA (Sigma‐Aldrich). DCFH‐DA (1 mmol/L) stock solution was diluted with DMEM to a concentration of 5 μmol/L. The cells were then incubated for 40 minutes at 37°C. The fluorescence intensity was measured after the cells were washed three times with phosphate buffered saline (PBS) using confocal microscopy (Carl Zeiss).

### 2.5 Measurement of mitochondrial membrane potential (Δψ)

Mitochondrial membrane potential was determined using JC‐1 (Sigma‐Aldrich), which is a cationic dye that exhibits potential‐dependent accumulation in mitochondria. First, H9c2 cells were seeded in confocal petri dishes. When the cells reached 70% confluency, they were treated according to the experimental design. JC‐1 working liquid was then prepared to a final concentration of 1 μg/mL. The JC‐1 working solution was added in a volume of 500 μL per well and incubated at 37°C for 20 minutes. The dye was then removed, and the wells were washed three times with PBS. The cells were observed using a confocal microscope (Carl Zeiss), and the ratio of red‐to‐green fluorescence intensities were analysed to determine the Δψ.

### 2.6 Relative mitochondrial (mt)DNA content measurements

Total DNA was extracted from H9c2 cells using an EZNA™ Tissue DNA Kit (Omega) according to the manufacturer's instructions. The mtDNA was measured using quantitative real‐time polymerase chain reaction (qRT‐PCR) analysis. Primers for cytochrome c oxidase subunit I (COXI) encoded by the heavy chain of mtDNA, NADH dehydrogenase subunit 1 (ND1), NADH dehydrogenase subunit 6 (ND6) and β‐globin were designed for qRT‐PCR by Generay. The primers are listed in Table 1.

**Table 1:** Primers for real‐time PCR

Gene	Forward primer (5'→3')	Reverse primer (5'→3')
GAPDH	AGCCAAAAGGGTCATCATCT	GGGGCCATCCACAGTCTTCT
ANP	ATCTGATGGATTCAAGAACC	CTCTGAGACGGGTTGACTTC
β‐MHC	CACTCCAGAAGAGAAGAACTCC	ATACTGTTGCCCACTTTGACT
BNP	ACAATCCACGATGCAGAAGCT	CGGCCTTGGTCCTTTGAGA
Sirt1	CACGATGGAGGGGCCGGACTCATC	TAAAGACCTCTATGCCAACACAGT
PGC1α	CCGAGAATTCATGGAGCAAT	TTTCTGTGGGTTTGGTGTGA
D‐loop	CAAACCTACGCCAAAATCCA	GAAATGAATGAGCCTACAGA
β‐globin	GCTTCTGACACAACTGTGTTCACTAGC	CACCAACTTCATCCACGTTCACC
cox1	CCACTTCGCCATCATATTCGTAGG	TCTGAGTAGCGTCGTGGTATTCC
ND6	TCACCCAGCTACCACCATCATTC	CACTGAGGAGTACCCAGAGACTTG
ND1	TCCTAACACTCCTCGTCCCTATTC	GGATGCCGTATGGACCTACAATG

### 2.7 The qRT‐PCR

Total RNA samples were prepared using a standard TRIzol protocol (Generay Biotech) and quantitatively measured using ultraviolet spectroscopy. Total RNA was amplified by qRT‐PCR using a two‐step RT‐PCR kit (Vazyme, Nanjing, China) according to the manufacturer's protocol. The qRT‐PCR analyses were repeated at least three times. The primers are listed in Table 1.

### 2.8 Western blot analysis

The H9c2 cells were collected in 1.5 mL microcentrifuge tubes, and 80 μL ice‐cold RIPA lysis buffer with phenylmethylsulfonyl fluoride and protein kinase inhibitors was added to the tubes, which were then centrifuged at 13800 *g* at 4°C for 10 minutes. The concentration of acquired protein was detected using a BCA kit (Beijing Dingguo Technology). Equivalent protein concentrations (40 µg) were loaded onto a 10% sodium dodecyl sulphate (SDS)‐polyacrylamide gel (running for about 90 minutes at 100 V) and then transferred to 0.45 µm polyvinylidene fluoride membrane (Millipore). The membranes were incubated in 5% non‐fat dry milk for 1 hour at room temperature and then incubated overnight at 4°C with one of the following primary antibodies: SIRT1 (Santa Cruz Biotechnology), glyceraldehyde 3‐phosphate dehydrogenase (GAPDH) (Affinity Biosciences), AMPK (Affinity Biosciences) or PGC‐1α (Abcam). The membranes were then incubated with secondary antibody (Affinity Biosciences) for 45 minutes at room temperature. After three washes in TBST buffer, antibody binding was detected using an ECL kit (Beijing Dingguo Technology). Protein band densities were quantified using a Gel‐Pro analyzer with version 4.0 software (Gel Analyzer; http://www.gelanalyzer.com/?i=1).

### 2.9 Immunohistochemistry

The cells were seeded in confocal petri dishes, and cultured at 37°C in 5% CO_2_ for 24 hours and then fixed with cold 4% paraformaldehyde for 10 minutes. After three washes in PBS, the cells were immersed in 1% Triton X‐100 for 5 minutes and then were incubated with anti‐SIRT1 antibody overnight at 4°C. The cells were then washed and incubated with tetramethylrhodamine isothiocyanate‐conjugated secondary antibody for 1 hour. After three washes in PBS buffer, cells were stained with 4, 6‐diamidino‐2‐phenylindole (DAPI) for 5 minutes and images were observed using a confocal fluorescence microscope (Carl Zeiss).

### 2.10 Statistical analysis

Data are presented as the mean ± standard error of the mean (SEM). Differences between groups were determined by one‐way analysis of variance followed by Tukey's post hoc test. Statistical significance was indicated by a value of *P* < .05. All statistical analyses were performed using GraphPad Prism, version 5.0 (GraphPad software).

## 3 RESULTS

### 3.1 STVNa promoted H9c2 cell viability following exposure to HG or ISO by decreasing the intracellular ROS accumulation

**Figure 1:**
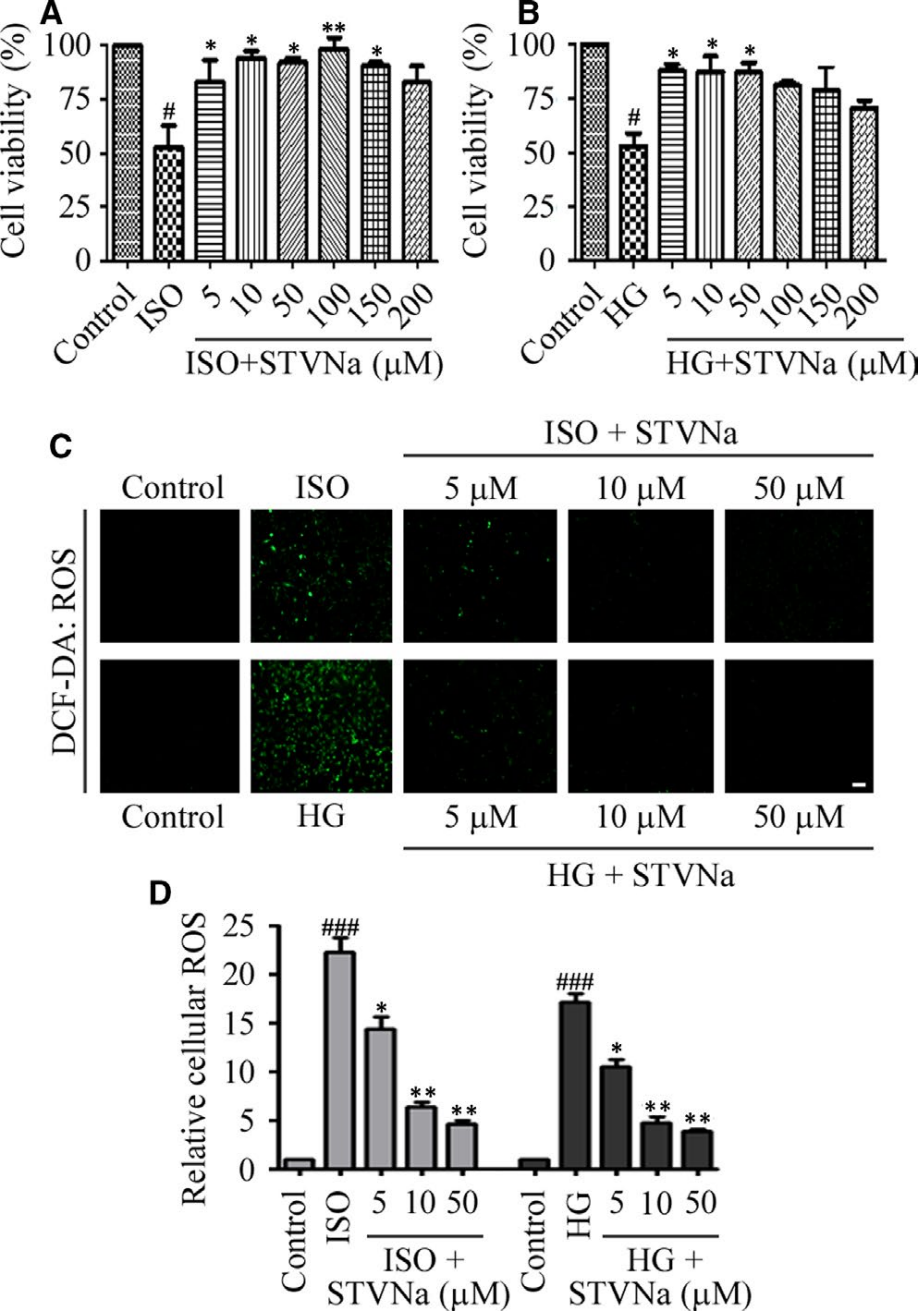
Isosteviol sodium (STVNa) inhibited isoprenaline (ISO)‐/high glucose‐induced cell viability loss and intracellular reactive oxygen species (ROS) accumulation. (A, B) Effect of STVNa on cell viability. H9c2 cells were treated with high glucose or (ISO) with or without STVNa for 48 h, and the cell viability in each group was assessed using the Cell Counting Kit‐8 assay. (C) Confocal images of ROS (Scale bar: 100 μm). (D) Graph of ROS levels. Images are representative of three individual experiments. Data are presented as the means ± SEM. ^#^ *P* < .05, ^###^ *P* < .001 vs the control group; ^*^ *P* < .05, ^**^ *P* < .01 vs the isoprenaline group

Compared with the control group, the viability of H9c2 cells treated with ISO or HG for 48 hours was significantly decreased (*P* < .05), as shown by the CCK‐8 staining assay. However, STVNa protected cells from damage caused by ISO or HG (Figure 1A,B). Specifically, the cell viability was increased following treatment with 5, 10 and 50 μmol/L STVNa, so we used 5, 10 and 50 μmol/L STVNa in subsequent studies. To determine whether STVNa decreased the intracellular ROS accumulation that occurred following exposure to ISO or HG, we examined the intracellular ROS levels. The results showed that H9c2 cells had a higher fluorescence intensity following treatment with ISO or HG, when compared with the control group (Figure 1C), showing that the ISO and HG groups produced high levels of ROS. However, the fluorescence intensity decreased significantly after 48 hours in the presence of STVNa, indicating that treatment with STVNa reduced intracellular ROS accumulation (Figure 1C,D).

### 3.2 STVNa protected H9c2 cells against HG‐ and ISO‐induced cardiomyocyte hypertrophy

**Figure 2:**
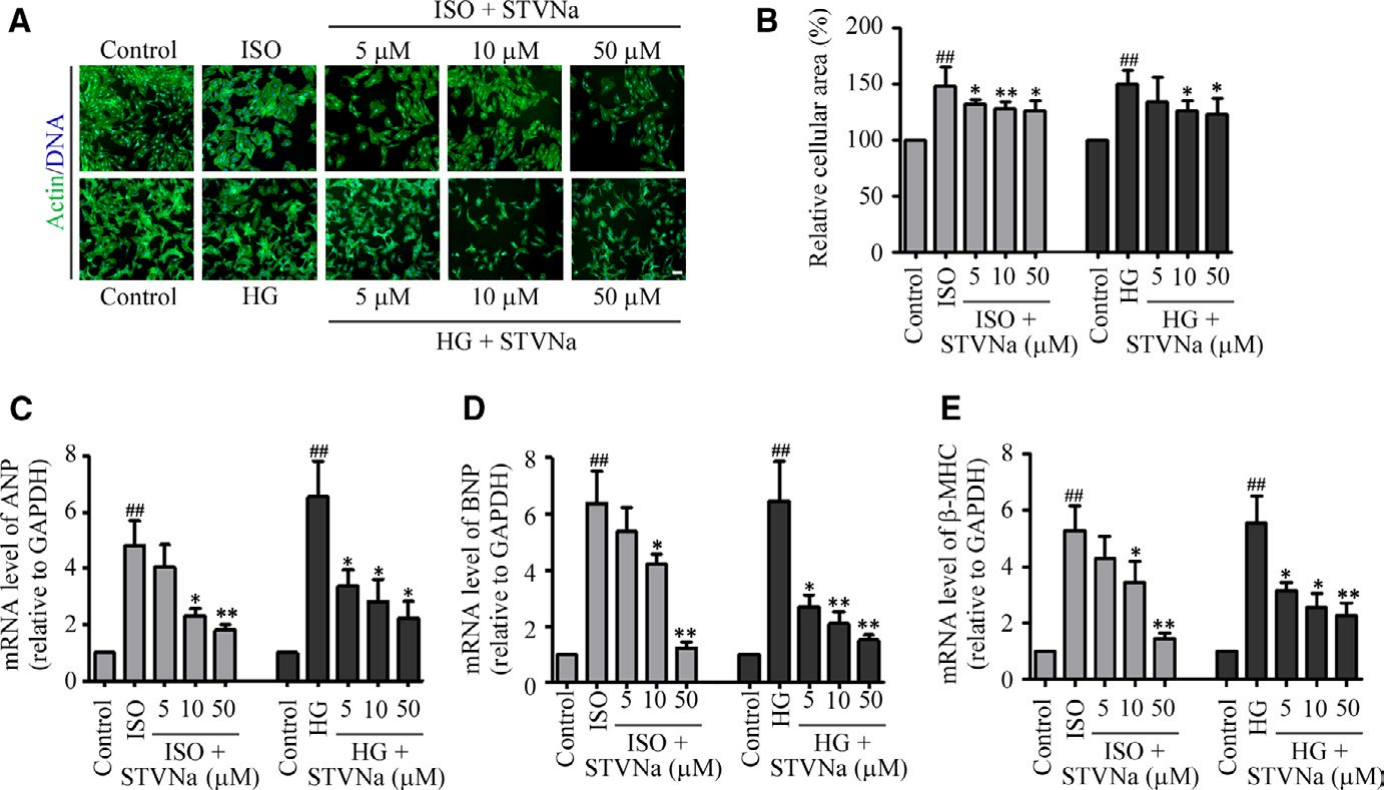
Isosteviol sodium (STVNa) protected H9c2 cells against HG‐ and ISO‐induced cardiomyocyte hypertrophy. (A) H9c2 cells were stained with phalloidin and 4, 6‐diamidino‐2‐phenylindole after treatment with 10 µmol/L ISO or 33.5 mmol/L glucose for 48 h (Scale bar: 100 μm), and a cell surface analysis of representative cells in each group was then performed and the results shown in (B). The qRT‐PCR analysis of the expressions of *ANP*, *β‐MHC* and *BNP* (C, D and E, respectively). Data are presented as the means ± SEM. ^##^ *P* < .01 vs the control group; ^*^ *P* < .05, ^**^ *P* < .01 vs the ISO or high‐glucose group

The H9c2 cells were treated with 33.5 mmol/L glucose or 10 µmol/L ISO for 48 hours. The results showed that HG and ISO significantly induced cardiomyocyte hypertrophy in H9c2 cells, as shown by the increase in cell surface area (Figure 2A,B). At the same time, the expression levels of *ANP*, *BNP* and *β‐MHC* were higher than those of the STVNa treatment groups (Figure 2C‐E). Their expression decreased significantly after 48 hours in the presence of STVNa, indicating that treatment with STVNa protected H9c2 cells from hypertrophy.

### 3.3 STVNa restored mitochondrial membrane potential (Δψ), maintained mitochondrial morphology, and increased mitochondrial biogenesis following exposure to HG or ISO

**Figure 3:**
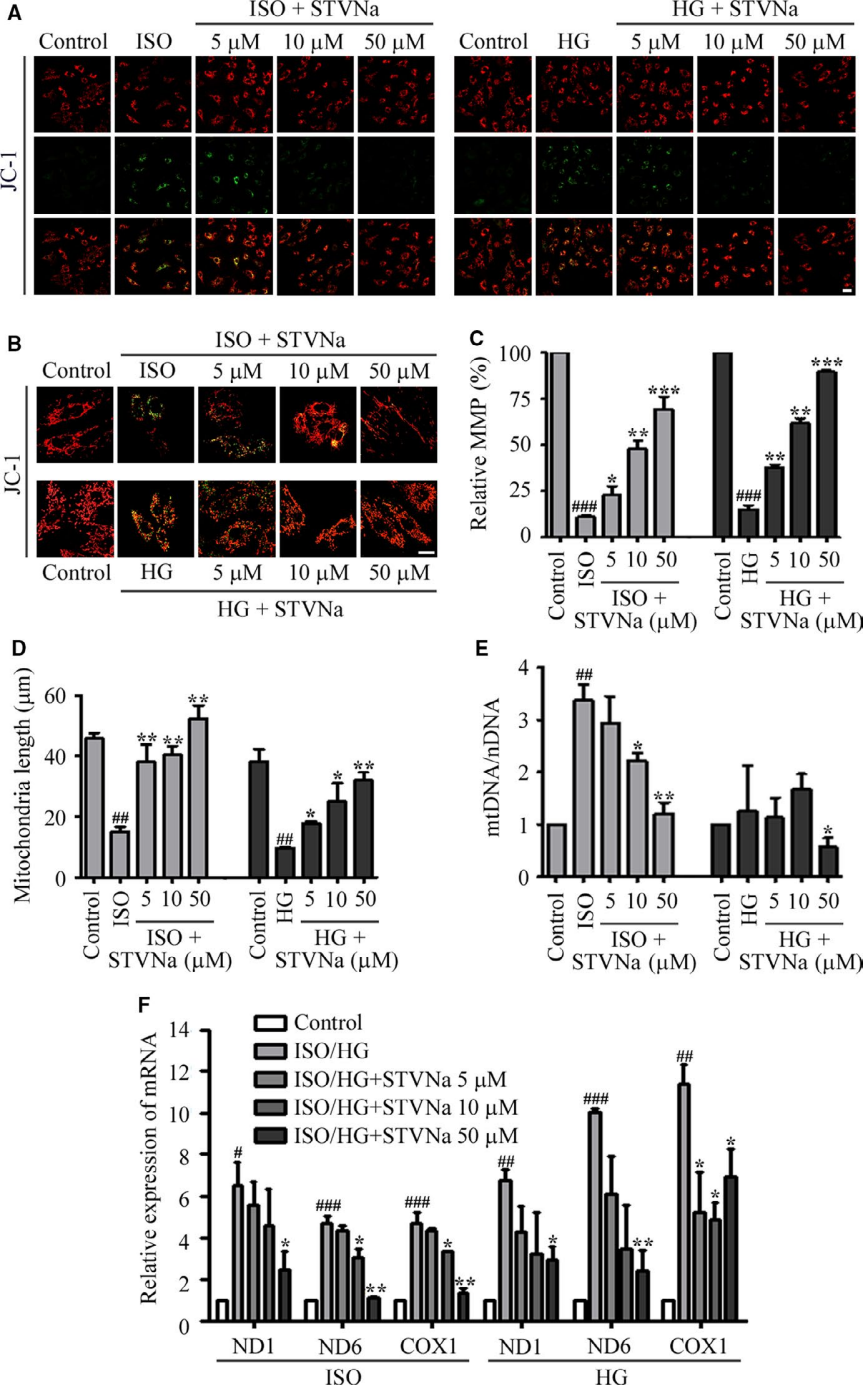
The effect of isosteviol sodium (STVNa) on mitochondrial membrane potential and mitochondrial morphology after exposure of cells to high glucose (HG) and isoprenaline **(**ISO) (A) Confocal images of mitochondrial potential following JC‐1 staining (scale bar: 50 μm). (C) Graph of the red‐to‐green (R/G) fluorescence intensities. (B) JC‐1 staining shows the tubular mitochondrial morphology (scale bar: 20 μm). The mitochondria became small and fragmented during HG and ISO exposures. STVNa maintained mitochondrial morphology to some extent. (D) Experiments were performed as in (B), and the length of the mitochondria was measured. (E) *COXI*, *ND1* and *ND6* mRNA levels. (F) The qRT‐PCR of the D‐loop area of the mitochondrial DNA. Data are presented as the mean ± SEM. ^#^ *P* < .05, ^##^ *P* < .01, ^###^ *P* < .01 vs the control group; ^*^ *P* < .05, ^**^ *P* < .01, ^***^ *P* < .01 vs the ISO or HG group

If mitochondrial function is disturbed, the cell viability decreases, so a decrease in Δψ is an indication of failing mitochondria. We therefore used the membrane sensitive dye, JC‐1, to stain cells to assess the effect of STVNa on Δψ. Figure 3A,C shows that the mitochondrial membrane potential of cells was reduced in the HG‐ and ISO‐treated groups. However, cells treated with 5, 10 or 50 μmol/L STVNa showed a partially recovered Δψ that occurred in a dose‐dependent manner (*P* < .05). To further study the protective effect of STVNa on mitochondrial function, we examined changes in mitochondrial morphology following exposure of H9c2 cells to HG or ISO. Figure 3B shows that the morphology of mitochondria became fragmented or smaller following HG or ISO exposure. The length of mitochondria was measured using ImageJ software (Figure 3D), indicating that STVNa significantly inhibited the effect of HG or ISO, to maintain the morphology of the mitochondria.

Mitochondrial biogenesis is stimulated by many factors, including exercise, diet, hormones and stress, which increase energy metabolism in cells. We therefore extracted DNA and mRNA from H9c2 cells and found that the mtDNA copy number and the levels of mtDNA transcripts (COXI, ND1 and ND6) were significantly lower in STVNa‐treated cells, when compared with those in the ISO or HG groups (Figure 3E,F).

### 3.4 The SIRT1/PGC‐1α signalling pathway was involved in the mechanism by which STVNa protected against cardiomyocyte dysfunction

**Figure 4:**
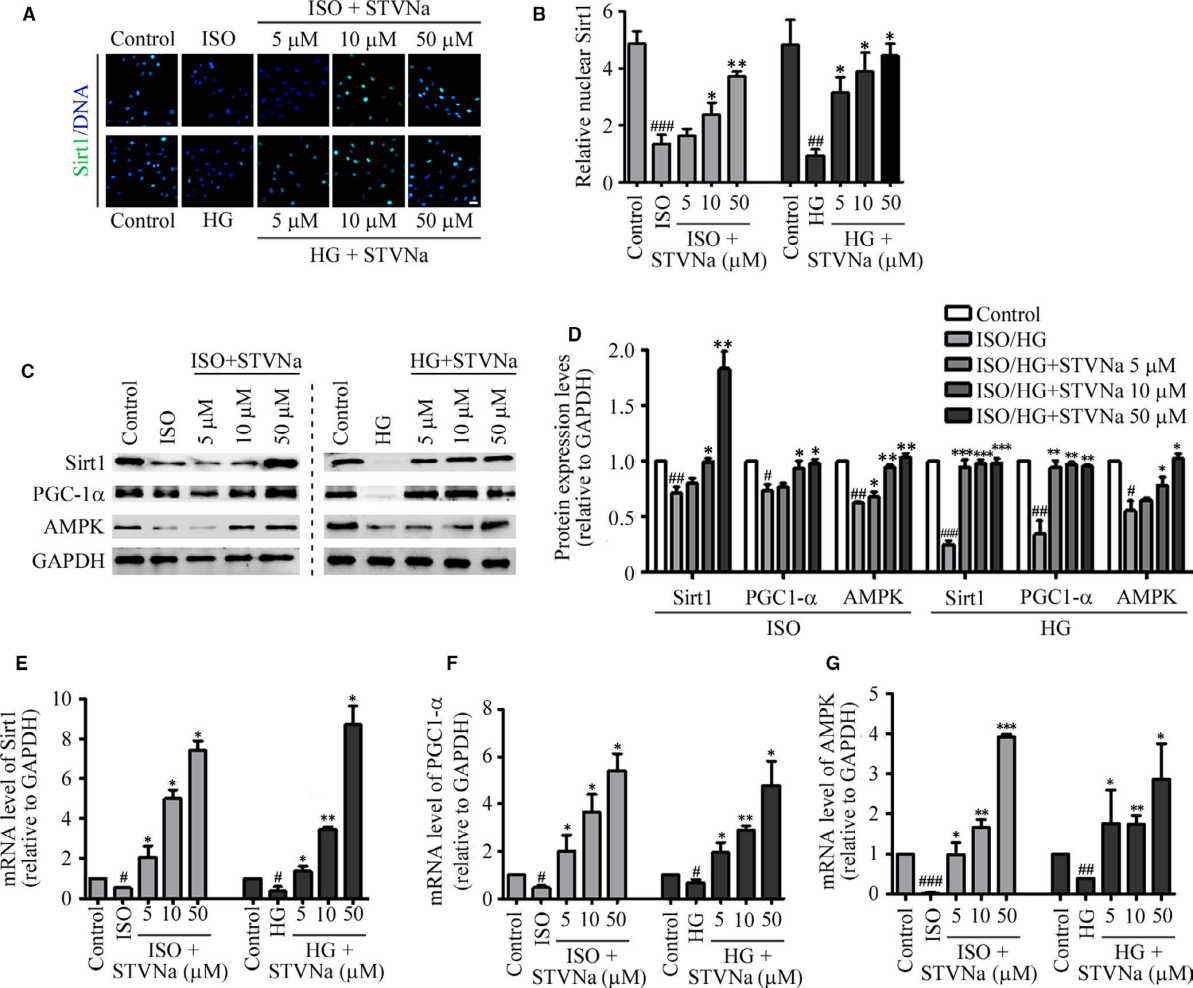
Isosteviol sodium stimulates silent information regulator transcript‐1 (SIRT1) activation following treatment with isoprenaline or high glucose in H9c2 cells. (A) Immunofluorescence detection of SIRT1 (scale bar: 50 μm). (B) Experiments were performed as in (A). The protein levels of SIRT1, PGC‐1α and glyceraldehyde 3‐phosphate dehydrogenase (GAPDH) were determined by Western blotting. (C) Experiments were performed as in (D). Quantitative analysis of SIRT1 and PGC‐1α normalized to GAPDH levels and expressed as relative fold changes versus the control group. The mRNA expressions of *SIRT1* and *PGC‐1α* were measured by qRT‐PCR (E, F and G). Data are presented as the mean ± SEM. ^#^ *P* < .05, ^##^ *P* < .01, ^###^ *P* < .01 vs the control group; ^*^ *P* < .05, ^**^ *P* < .01, ^***^ *P* < .001 vs the isoprenaline or high‐glucose group

SIRT1 plays an important regulatory role in mitochondrial biogenesis by activating PGC‐1α. To examine potential mechanisms through which STVNa modulates signalling pathways, we assessed the protein levels of SIRT1 and PGC‐1α using immunofluorescent staining and Western blot assays. The results indicated that the nuclear protein levels of SIRT1 and PGC‐1α were significantly increased after treatment with STVNa in cells exposed to HG or ISO (Figure 4A,B). However, the regulatory effects of STVNa on the protein expressions of SIRT1 and PGC‐1α in the HG and ISO models and the dosage effects of STVNa in these two groups were different (Figure 4C,D). We also showed that the mRNA expressions of SIRT1, PGC‐1α and AMPK were significantly increased by treatment with STVNa (Figure 4E‐G). These results suggested that the cardiomyocyte protective effects of STVNa were associated with the up‐regulation of the SIRT1/PGC‐1α signalling pathway. However, the mechanism of action of STVNa may be different in these two models.

### 3.5 The effect of the SIRT1 activator, resveratrol, and the SIRT1 inhibitor, Ex527, on SIRT1 activity in H9c2 cells

**Figure 5:**
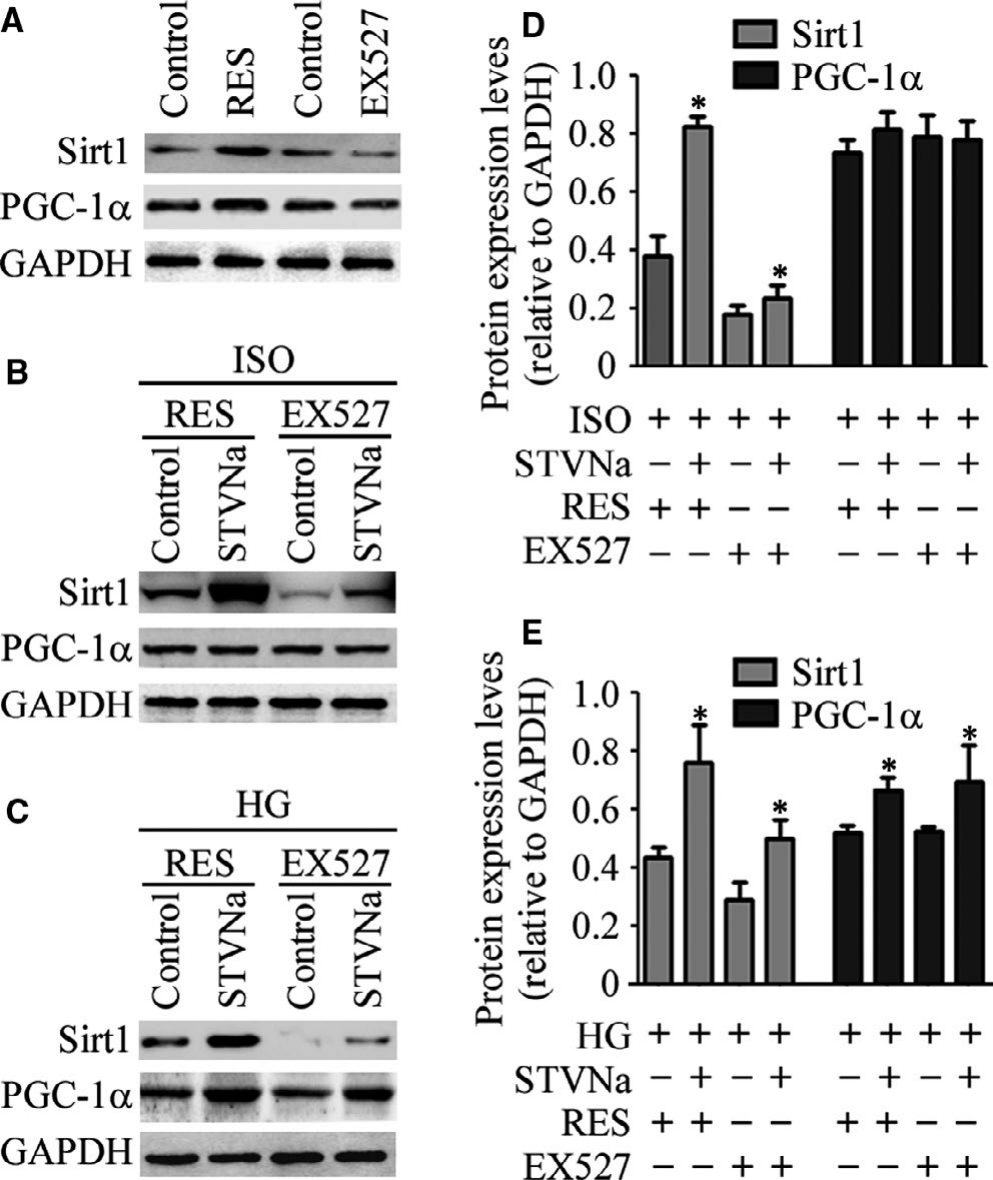
The effect of the silent information regulator transcript‐1 (SIRT1) activator, resveratrol, and the SIRT1 inhibitor, Ex527, on SIRT1 activity in H9c2 cells. (A) H9c2 cells were treated with RES (30 μmol/L) or Ex527 (10 μmol/L) for 48 h, and the protein levels of SIRT1, PGC‐1α and glyceraldehyde 3‐phosphate dehydrogenase were determined by Western blot analysis. (B and C) H9c2 cells were incubated with ISO or HG in the presence or absence of STVNa (10 μmol/L) for 48 h. EX‐527 (10 μmol/L) or RES (30 μmol/L) was added 1 h before STVNa treatment. The protein levels of SIRT1, PGC‐1α and glyceraldehyde 3‐phosphate dehydrogenase were determined by Western blot analysis. (D and E) Experiments were performed as in (B and C), levels of SIRT1 and PGC‐1α. Protein levels are relative to the control group. All values are expressed as the mean ± SEM. ^*^ *P* < .05 vs the control group

Resveratrol (3,5,4′‐trihydroxy‐trans‐stilbene) is a polyphenol phytoalexin present in a variety of plant species and has drawn much attention due to its beneficial effects.^15^ Additionally, recent studies have shown that resveratrol could improve the expression of SIRT1 and activate the AMPK‐SIRT1‐autophagy signal pathway.^16^ In the present study, resveratrol (RES) was used as an agonist of SIRT1 and Selisistat (EX‐527) was an inhibitor of SIRT1 enzymatic activity. To further ascertain the involvement of the SIRT1/PGC‐1α signalling pathway in the mechanism of STVNa protection against cardiomyocyte dysfunction, we determined the influence of the SIRT1 activator, resveratrol (RES), and the SIRT1 inhibitor, Ex527, on SIRT1 activity. H9c2 cells were incubated with ISO or HG in the presence or absence of STVNa (10 μmol/L). EX‐527 (10 μmol/L) or RES (30 μmol/L) was added 1 hour before STVNa treatment. Figure 5 shows that pre‐treatment with RES prevented ISO‐ and HG‐induced SIRT1 down‐regulation. In addition, the expression of SIRT1 was much higher when pre‐treatment combined with RES and STVNa than pre‐treatment with RES alone. It indicated that STVNa could further activate SIRT1 expression and the downstream signal pathway (Figure 5A‐C). SIRT1 was suppressed by EX527, and the effect of STVNa regulating on SIRT1 was significantly attenuated when H9c2 cells were treated with EX527 and STVNa (Figure 5D,E). Interestingly enough, the expression trend of PGC‐1α was consistent with SIRT1 in the HG group rather than in the ISO group. Notably, compared with the HG group, the expression of SIRT1 in H9c2 cells did not affect the expression of PGC‐1α following treatment with EX‐527 or RES (Figure 5). These results suggested that there may be different mechanisms of action of STVNa in these two models. The different mechanisms may be associated with the different mechanisms of regulation of STVNa on the expression and the post‐translational modification of PGC‐1α. STVNa may activate the expression levels of SIRT1 and PGC1‐1α during HG treatment, whereas SIRT1 may activate PGC‐1α by regulating deacetylation instead of simply up‐regulating its protein levels when H9c2 cells treated with STVNa were exposed to EX‐527 and RES in the ISO group.

## 4 DISCUSSION

Previous studies have demonstrated the cardioprotective effects of STVNa. For example, isosteviol has been shown to ameliorate diabetic cardiomyopathy in rats,^17^, ^18^ and STVNa has been shown to protect H9c2 cells against myocardial ischaemia reperfusion injury.^19^ However, these studies have not identified the specific molecular mechanisms by which STVNa acts at the cellular level. Our study showed that STVNa exerted a cardioprotective effect in ISO‐/HG‐induced myocardial hypertrophy through a mitochondrial‐mediated signalling pathway.

Myocardial hypertrophy is the pathological manifestation of hypertensive heart disease, which is an independent risk factor leading to an increase in cardiovascular disease morbidity and mortality. HG and ISO are traditional methods used to induce cardiac hypertrophy. Previous studies have reported that cell models are successful when the concentration of ISO is 10 μmol/L^20^ and when the glucose concentration is 30.5 nmol/L. We therefore first analysed the cytotoxic effect of STVNa with ISO/HG on H9c2 cells. Our results showed that STVNa had no toxic effect on cells when the concentration was <200 µmol/L in the presence of HG or ISO. If ROS generation exceeds its elimination, it results in cell damage that ultimately leads to death. We found that exposure to HG and ISO induced a significant increase in ROS. However, treatment with STVNa reduced intracellular ROS levels and protected the mitochondria.

Mitochondria are the major sites of ATP synthesis, involving the process of oxidative phosphorylation. Mitochondrial health must be monitored carefully because mitochondria are sensitive to the major metabolic pathways of the cell,^21^ and dysfunctions can cause cell death. Mitochondria also mediate amino acid biosynthesis, fatty acid oxidation, steroid metabolism, calcium homoeostasis, and ROS production and detoxification.^22^ Inhibition of the respiratory chain, a decrease in ATP production, loss of the mitochondrial membrane potential and a reduction in mtDNA copy number are considered examples of mitochondrial dysfunctions.^23^ Mitochondria are dynamic bodies that constantly divide and fuse within the cell, depending on environmental demands.^24^ These processes can facilitate the formation of new mitochondria, repair of defective mtDNA through mixing and redistribution of the mitochondria to sites requiring high energy production.^25^, ^26^ The results of the present study showed that STVNa protected the mtDNA from damage, showing that treatment with STVNa increased mitochondrial biogenesis in H9c2 cells.

The SIRT1/PGC‐1α pathway is an important signalling pathway in myocardial hypertrophy. SIRT1 can affect mitochondrial function and regulate energy metabolism by increasing the activity of PGC‐1α SIRT1. Expression levels in spontaneously hypertensive rats have been reported to be higher than those in normal rats, and the occurrence of hypertensive left ventricular hypertrophy has been positively correlated with *SIRT1* mRNA levels in myocardial tissue.^27^ SIRT1 has a complex effect on eccentric hypertrophy. Planavila et al reported that SIRT1 inhibited the hypertrophy of myocardial cells caused by phenylephrine and suggested that this effect was related to peroxisome proliferator‐activated receptor (PPAR).^28^ AMPK can also phosphorylate SIRT1 to regulate its activity or expression.^29^, ^30^ AMPK plays a role in multiple intracellular biological functions in ischaemia, and levels of SIRT1 and AMPK are associated with each other. PGC‐1α acts as a co‐ordinator of SIRT transcription factors, although at the same time, SIRT1 can also increase PGC‐1α activity to affect mitochondrial function and regulate energy metabolism.^31^, ^32^

Many studies have found that SIRT1 occurs upstream of ROS. We could not directly infer that ROS production was downstream of SIRT1, but our results indicated that STVNa increased the level of SIRT1, and activation of SIRT1 led to increased ROS damage and oxidative stress. We therefore concluded that STVNa reduced oxidative stress through the SIRT1/PGC‐1α pathway. The SIRT1/PGC‐1α signalling pathway plays an important role in the regulation of oxidative stress. Studies related to oxidative stress have shown that this pathway exists in different cell types, but it has rarely been studied in H9c2 cells. Our results also showed that HG and ISO inhibited SIRT1 and PGC‐1α, and STVNa significantly activated SIRT1 and PGC‐1α.

**Figure 6:**
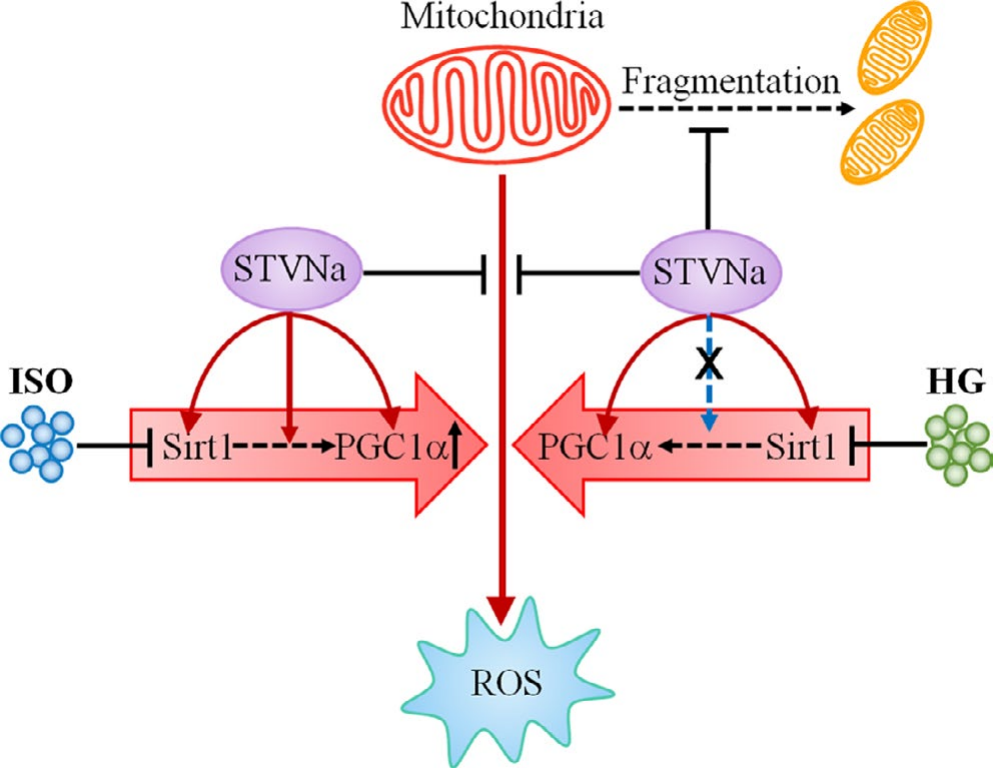
Mechanistic illustration of the effects of STVNa on ISO‐ and HG‐induced cardiac hypertrophy. STVNa elevates SIRT1 and PGC‐1α expression, inhibits cellular ROS overload and ROS‐induced cell mitochondrial fission, thus restoring MMP and mitochondrial morphology. HG, high glucose; ISO: isoprenaline; PGC‐1α, peroxisome proliferator‐activated receptor gamma coactivator 1‐alpha; ROS, oxygen species; Sirt1, silent information regulator transcript‐1; STVNa, isosteviol sodium

The mechanism by which HG and ISO induce myocardial hypertrophy differs. ISO, as a non‐selective beta agonist, increases heart rate, cardiac contractile force and comfortable tension when it acts on the β1 adrenoceptor. ISO also induces diastole in blood vessels and the trachea by acting on the β2 adrenoceptor.^33^, ^34^ Chronic β1 adrenoceptor stimulation further induces myocardial hypertrophy. However, HG is also commonly used as a model of myocardial hypertrophy. The mechanism by which it induces myocardial hypertrophy differs from ISO. HG causes high blood glucose toxicity, which can induce oxidative stress, and increases the formation of ROS and compensatory myocardial hypertrophy to accelerate the necrosis and apoptosis of myocardial cells, resulting in a decrease of myocardial cell number. HG causes myocardial cell injury and ultimately induces diabetic cardiomyopathy and dilated cardiomyopathy, cardiac arrhythmia, heart failure, and even sudden death.^17^, ^35^, ^36^ These differences may be the reason that the regulatory effects of STVNa on the protein expressions of SIRT1 and PGC‐1α in HG and ISO models and the dosage effects of STVNa in these two groups were different in this study. Overall, these results suggested that the molecular mechanisms of STVNa in the ISO and HG models may differ (Figure 6).

In the present study, we showed a role for STVNa in inhibiting HG‐ and ISO‐induced oxidative stress and mitochondrial damage in H9c2 cells, and found that it attenuated oxidative stress and restored the mitochondrial membrane potential to restore mitochondrial morphology. Furthermore, we found that STVNa reduced HG‐ and ISO‐induced damage via activation of the AMPK and SIRT1/PGC‐1α signalling pathways. Our results indicated that the SIRT1/PGC‐1α pathway plays a role in protecting the heart and that it is associated with a combined and balanced effect on mitochondrial biogenesis and function. This is the first time the protective effect of STVNa in H9c2 cells has been demonstrated to occur via regulation of the SIRT1/PGC‐1α pathway. These findings suggested a positive effect of STVNa on mitochondrial functional enhancement in H9c2 cells via activation of the SIRT1/PGC‐1α pathway. Furthermore, the mechanisms of action of STVNa may be different between ISO and HG models, and the different mechanisms may be associated with the different regulation of STVNa on the activation of PGC‐1α. Taken together, these findings may help us identify the molecular mechanisms of STVNa during its cardioprotective effects.

## CONFLICT OF INTEREST

The authors declare that they have no competing interests.

## AUTHOR CONTRIBUTIONS

**Ying Mei:** Data curation (equal); Writing‐original draft (equal). **Bo Liu:** Formal analysis (equal); Methodology (supporting). **Hao Su:** Data curation (equal). **Hao Zhang:** Data curation (equal); Writing‐review & editing (supporting). **Fei Liu:** Data curation (equal); Formal analysis (supporting). **Qingjin Ke:** Formal analysis (equal); Software (equal). **Xiaoou Sun:** Conceptualization (lead); Project administration (equal); Writing‐review & editing (lead). **Wen Tan:** Funding acquisition (equal); Project administration (equal).

## Data Availability

The data that support the findings of this study are available on request from the corresponding author. The data are not publicly available due to privacy or ethical restrictions.
